# Effectiveness and feasibility of an interprofessional training program to improve patient safety—A cluster-randomized controlled pilot study

**DOI:** 10.3389/fpsyg.2023.1186303

**Published:** 2023-11-07

**Authors:** Mirjam Körner, Julia Dinius, Nicole Ernstmann, Lina Heier, Corinna Bergelt, Antje Hammer, Stefanie Pfisterer-Heise, Levente Kriston

**Affiliations:** ^1^Medical Faculty, Institute of Medical Psychology and Medical Sociology, University of Freiburg, Freiburg, Germany; ^2^Department of Health Professions, Competence Centre Interprofessionalism, Bern University of Applied Sciences, Bern, Switzerland; ^3^Institute for Patient Safety, University Hospital Bonn, Bonn, Germany; ^4^Chair of Health Services Research, Faculty of Medicine and University Hospital Cologne, Institute of Medical Sociology, Health Services Research and Rehabilitation Science, University of Cologne, Cologne, Germany; ^5^Department for Psychosomatic Medicine and Psychotherapy, Center for Health Communication and Health Services Research, University Hospital Bonn, Bonn, Germany; ^6^Department of Medical Psychology, University Medicine Greifswald, Greifswald, Germany; ^7^Department of Medical Psychology, University Medical Center Hamburg-Eppendorf, Hamburg, Germany

**Keywords:** patient safety, teamwork, patient involvement, error management, healthcare, training, cluster randomized controlled study

## Abstract

**Introduction:**

Interprofessional healthcare teams are important actors in improving patient safety. To train these teams, an interprofessional training program (IPTP) with two interventions (eLearning and blended learning) was developed to cover key areas of patient safety using innovative adult learning methods. The aims of this study were to pilot test IPTP regarding its effectiveness and feasibility. The trial was registered with DRKS-ID: DRKS00012818.

**Methods:**

The design of our study included both a pilot investigation of the effectiveness of the two interventions (eLearning and blended learning) and testing their feasibility (effectiveness-implementation hybrid design). For testing the effectiveness, a multi-center cluster-randomized controlled study with a three-arm design [intervention group 1 (IG1): eLearning vs. intervention group 2 (IG2)]: blended learning (eLearning plus interprofessional in-person training) vs. waiting control group (WCG) and three data collection periods (pre-intervention, 12 weeks post-intervention, and 24 weeks follow-up) was conducted in 39 hospital wards. Linear mixed models were used for the data analysis. The feasibility of IPTP was examined in 10 hospital wards (IG1) and in nine hospital wards (IG2) using questionnaires (formative evaluation) and problem-focused interviews with 10% of the participants in the two intervention groups. The collected data were analyzed in a descriptive exploratory manner.

**Results:**

Pilot testing of the effectiveness of the two interventions (eLearning and blended learning) showed no consistent differences between groups or a clear pattern in the different outcomes (safety-related behaviors in the fields of teamwork, error management, patient involvement, and subjectively perceived patient safety). Feasibility checks of the interventions showed that participants used eLearning for knowledge activation and self-reflection. However, there were many barriers to participating in eLearning, for example, lack of time or access to computers at the ward. With regard to in-person training, participants stated that the training content sensitized them to patient-safety-related issues in their everyday work, and that awareness of patient safety increased.

**Discussion:**

Although the interventions were judged to be feasible, no consistent effects were observed. A possible explanation is that the duration of training and the recurrence rate may have been insufficient. Another conceivable explanation would be that participants became more sensitive to patient safety-critical situations due to their knowledge acquired through the IPTP; therefore, their assessment post-intervention was more critical than before. In addition, the participants reported high pre-measurement outcomes. Future studies should examine the evidence of the intervention within a confirmatory study after adapting it based on the results obtained.

## 1. Introduction

Patient safety is a cornerstone of healthcare delivery. It aims to ensure that patients receive the best possible care free from preventable harm. It refers to the prevention of errors and adverse events in healthcare, and encompasses a wide range of issues and concerns, including preventing errors in the administration of medications and in medical procedures, and reducing the risk of infection (Mitchell, 2008). Patient safety is an interprofessional effort that requires the active engagement of different healthcare providers (interprofessional teams), patients, and their families; effective communication and collaboration among them is essential for patient safety (Berry et al., 2020; Dinius et al., 2020). Additionally, patient safety also involves educating patients and their families about their own health and healthcare and encouraging them to actively participate in their caregiving (Wright et al., 2016; Wu and Busch, 2019). Proactive measures for identifying and addressing any potential hazards are also important to reduce the risk of errors and adverse events (Spurgeon et al., 2019).

Improving patient safety is a political goal in healthcare globally (World Health Organization [WHO], 2021). Interprofessional healthcare teams are the relevant actors in achieving this goal. Internationally, especially in North America, training programs exist, but often focus on one specific patient safety topic, for example, the prevention of falls, and infection control. Furthermore, they are often developed for healthcare professionals but do not cover the interprofessional aspects of working together in a team (Reeves et al., 2017; Dinius et al., 2019; Amaral et al., 2023). An exception is the TeamSTEPPS^®^ 2.0 training program, which is a comprehensive, evidence-based, and commonly used program in North America that was brought to Germany at the same time, but independently of our study.^1^

A meta-analysis by Schmutz et al. (2019) showed that interprofessional teamwork has a medium-sized effect on team performance. Therefore, healthcare organizations should bolster interprofessional teamwork to enhance patient safety (Schmutz et al., 2019). Furthermore, teamwork interventions have a positive and significant medium-sized effect on teamwork and team performance (McEwan et al., 2017). In turn, teamwork is associated with improved patient outcomes and increased patient safety (Dinius et al., 2020).

There are different approaches to improve patient safety. Previous research suggests that interventions such as trainings effectively improve teamwork, patient engagement, support of cultural changes, and information technology to subsequently reduce medical errors (Woodward et al., 2010; Amaral et al., 2023). A culture of safety can be built through open discussions regarding adverse events, errors, and their consequences for quality of care (Hofinger, 2009). Furthermore, patients should play an active role in error prevention (Schwappach, 2010), which can be achieved by intensifying patient participation, such as, by involving patients in patient safety management (Wright et al., 2016), by informing patients and encouraging them to participate, providing necessary information promptly and comprehensibly, and enhancing their ability to identify patient safety incidents. However, patient participation in patient safety is lacking in clinical practice. Training programs are required to create a culture of safety that includes patient participation in healthcare processes (Sahlström et al., 2019). Such a culture, in which all professionals of the interprofessional team and patients are seen as equal partners, is still missing. Since the participation of both patients and healthcare professionals is a major factor in high-quality and safe patient-centered care, addressing these topics in training may be advantageous (Quaschning et al., 2013; Hwang et al., 2019).

To address this gap, the KOMPAS project (KOMPAS = German acronym for “Development and evaluation of a complex training program to improve patient safety”) developed and implemented an interprofessional training program (IPTP) with two interventions. These utilized eLearning as well as blended learning, with both covering three key areas of patient safety (teamwork, patient involvement, and error management). The aims of this study were to pilot-test the IPTP by comparing the two types of interventions (eLearning and blended learning) with a waiting control group. The study also aimed to pilot test the effectiveness and the feasibility of these interventions.

The improvement in safety-related behavior regarding teamwork, patient involvement, and error management, as well as subjectively perceived patient safety, was expected to be significantly higher in intervention group 1 (IG1: eLearning) and intervention group 2 (IG2: blended learning: eLearning and interprofessional in-person training) than in the waiting control group (WCG). The greatest improvement was predicted for IG2.

In the course of piloting the effectiveness of the interventions, the following research question and hypothesis were pursued:

1. Do the interventions improve safety-related behavior?

H1: The improvement of safety-related behavior in IG1 and IG2 is significantly higher than in WCG.

In the eLearning course, the participants worked individually on the theoretical foundations for the three key areas. Due to the additional interprofessional in-person training, the participants tried out and consolidated their knowledge. This led us to the following hypothesis:

H2: The greatest improvement in safety-related behavior is in IG2.

2. Do the interventions improve subjectively perceived patient safety?

H3: The improvement of subjectively perceived patient safety in IG1 and IG2 is significantly higher than in WCG.

H4: The greatest improvement of subjectively assessed patient safety is in IG2.

In course of evaluating the feasibility of the interventions the following research questions were investigated:

3. How do trained professionals assess the feasibility of eLearning and interprofessional in-person training?

4. Which facilitators and barriers to implementing the interventions can be identified?

## 2. Materials and methods

For the following description of the study design and outcomes, intervention, data collection process, and data analysis, the CONSORT guidelines extended for cluster randomized trials (Campbell et al., 2012) and randomized pilot and feasibility trials (Eldridge et al., 2016) were used as standards.

### 2.1. Study design and outcomes

The design included both a pilot investigation of the effectiveness of the interventions and testing their feasibility. Accordingly, our investigation can best be labeled as a pilot study using a hybrid effectiveness-implementation design (Curran et al., 2012).

#### 2.1.1. Pilot test of the effectiveness

To pilot test the effectiveness of the IPTP, a multi-center cluster-randomized controlled study with a three-arm design [intervention group 1 (IG1)]: eLearning vs. intervention group 2 (IG2): blended learning vs. waiting control group (WCG) and three data collection periods (t1: pre-intervention, t2:12 weeks post-intervention, and t3:24 weeks follow-up) was conducted at three study sites (Freiburg, Hamburg, Bonn) between 2017 and 2020. Randomization took place at the ward/team level (clusters). The teams were randomly assigned to the three study arms by an independent statistician, who was not involved in the recruitment or implementation of the intervention based on a computer-generated randomization sequence with a 1:1:1 treatment allocation ratio. For detailed information see study protocol by Dinius et al. (2019).

**FIGURE 1:**
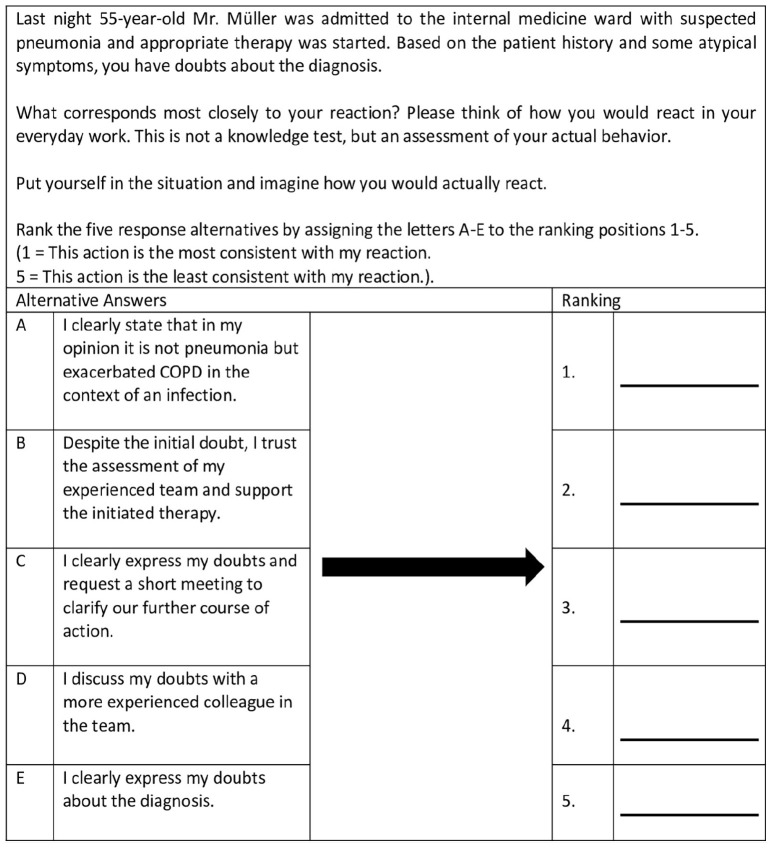
Vignette on teamwork. Participants had to rank the five response alternatives by assessing the letters A–E to the ranking positions 1–5.

The outcome subjectively perceived patient safety was assessed with a single item from the German Hospital Survey on Patient Safety (HSPSC-D) (Gambashidze et al., 2017, value range five level: insufficient, poor, acceptable, very good, and excellent). To measure the safety-related behavior regarding teamwork, error management, and patient involvement situational judgment tests (SJT) (McDaniel et al., 2007; Lievens et al., 2009; Christian et al., 2010) consisting of three self-developed vignettes aligned with the three topics were conducted. The situations depicted in the vignettes were exemplary for situations with special relevance for patient safety (e.g., patient mix-up, adverse drug events, team communication about a doubtful diagnosis). In all three vignettes, the description of the situation was followed by the instruction “Put yourself in the situation and imagine how you actually would react.” The answer categories were developed by the research team and reviewed in an interprofessional expert workshop. Participants were instructed to rank the five response alternatives by assessing the letters A-E depending on which behavior would be best and which behavior would be worst in their perspective. The ranking positions ranged from 1 (this action is the most consistent with my reaction) to 5 (this action is the least consistent with my reaction). The ideal sequence, based on results of the expert workshop, was scored with 30 points (= 4 × 4 + 3 × 3 + 2 × 2 + 1 × 1 + 0 × 0). The worst sequence was scored with 10 points (= 4 × 0 + 3 × 1 + 2 × 2 + 1 × 3 + 0 × 4). For better interpretation of the data, scores were transformed from 0 to 100. Figure 1 shows an example of a vignette on teamwork.

Owing to the longitudinal study design, the above-mentioned outcomes were assessed at all three data collection periods. The questionnaire during the first data collection period also included sociodemographic information (age, gender, profession, leadership position, duration of hospital affiliation and occupational affiliation in years).

#### 2.1.2. Pilot test of the feasibility

To pilot test the feasibility of the interventions short self-developed written surveys (formative evaluation) and problem-focused individual interviews were used. Participants evaluated eLearning in an online written survey using seven items to assess satisfaction, acceptance, and user-friendliness on a scale of 1 (strongly disagree) to 5 (strongly agree) using adapted items from the two standardized questionnaires: System Usability Scale (SUS) (Brook, 1996) and the measure success inventory (MEI) (Kauffeld et al., 2009). The items are listed in Table 4. The evaluation could be completed after finishing the eLearning intervention.

A short self-developed paper-based survey was distributed by the instructors after finishing the interprofessional in-person training. Participants rated in-person training using nine items on a scale of 1 (strongly disagree) to 5 (strongly agree). The interventions were evaluated with respect to satisfaction and acceptance. The items are listed in Table 5.

Additionally, each intervention also received an overall grade from the participants using the German school grading system from 1 (very good) to 6 (deficient).

Problem-focused individual interviews with 10% of the participants in the intervention groups (IG1 and IG2) were conducted as part of the post-measurement. In these interviews, facilitators, and barriers to implementing training in the participants’ daily work routines were explored. Questions related to context, design, and comprehensibility were also included.

### 2.2. Intervention

Our intervention consists of two components: eLearning and interprofessional in-person training. In recent years, eLearning has become a standard methodological approach in teaching (George et al., 2014). It offers participants flexibility in terms of place and time of learning. From a learning theory perspective, the same basic concepts are used as they are found in other forms of teaching. Our training based on the following pertinent learning theories:

1. Adult Learning Theory (Knowles, 1984): We designed for the eLearning interactive, case-based, and experiential learning opportunities that actively engage participants and promote critical thinking and problem-solving skills.
2. Cognitive Load Theory (Sweller, 1988) means simplifying complex concepts, providing clear and concise instructions, and using multimedia and interactive elements effectively to manage cognitive load and enhance learning outcomes in both components of our intervention.
3. Social Learning Theory (Bandura, 1977) to conceptualize the interprofessional in-person training. This theory underscores the importance of role modeling and peer learning. In the interprofessional in-person training the participants can learn from experts, engage in discussions and collaborative learning.

For developing the IPTP we considered the key patient safety principles, such as human factors, systems approach, teamwork, communication and error reporting (Lee et al., 2022). Based on the learning objectives of the Patient Safety Curriculum Guide of the World Health Organization [WHO] (2011), the Patient Safety Curriculum Guide of the Patient Safety Action Alliance (Aktionsbündnis Patientensicherheit, 2022), and a focus group study of our research group (Dinius et al., 2017), an IPTP was developed. It consists of two parts: a 3-h eLearning and a three and a half hour interprofessional in-person training. In both the eLearning and the in-person training three learning modules (topics: teamwork, error management, patient involvement), which are essential for patient safety, were conducted. These three topics are interrelated and associated with learning objectives for individual healthcare professionals.

**FIGURE 2:**
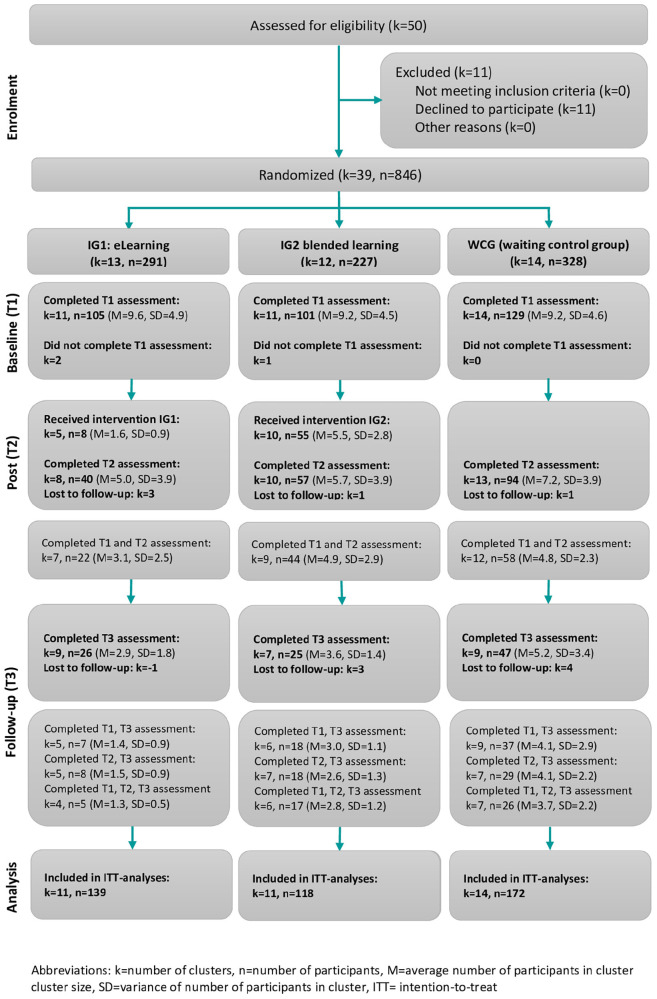
Consort flow diagram.

For the development of the KOMPAS eLearning, ELPAS (eLearning Patient Safety), which is used at the Albert-Ludwigs University of Freiburg for training medical students, served as a basis. All modules were programmed with Adobe Captivate and were made available to the participants password-protected via the learning platform Weiterbildungs-Ilias at the Albert-Ludwigs-University of Freiburg. Meanwhile, eLearning is open access.^2^ A video served as an introduction to eLearning, in which participants were introduced to the central learning objective -the improvement of patient safety–and were familiarized with the use of eLearning. This was followed by three learning modules on the key aspects of patient safety. Each module started with an introductory video on the respective topic followed by the related content, which was presented in smaller submodules (completion time 5–10 min) (Figure 2). Participants had the possibility to pause eLearning at any time and continue at a later point in time. The end of each learning module was a short summary of the content (take-home messages).

To obtain the continuing education points (CME points for physicians or continuing education points for voluntarily registered professional nurses) after completing the eLearning modules, all participants completed a final test with 39 questions (13 questions per module). There were five alternative answers to each question, of which one answer was correct. The pass mark was 70%. All participants were given two attempts to pass the final test.

Corresponding to the eLearning design, the interprofessional in-person training also consisted of three modules. Interactive video analysis was conducted in the teamwork module. In this video, the patient is being returned to spontaneous circulation by a team. The participants’ task was to analyze the interprofessional teamwork of the resuscitation team and develop suggestions for improvement based on their analysis. This was followed by group work in which the following questions were to be worked on: “Are there specific situations in which safety concerns are not addressed?” as well as “Are there typical strategies that team members use to avoid having to address safety concerns?” At the end of the teamwork module, the participants were instructed to put themselves in the role of a member of the resuscitation team from the video and to practice the “Speak Up” method. A prepared flipchart of the theory and formulation possibilities of Speak Up was available as an aid.

In the error management module, the participants were given a critical incident reporting system (CIRS) case, which they had to analyze in their respective small groups with regard to cause and effect. The developed worksheet “Identifying Factors Promoting Errors,” which shows examples at different system levels, such as patient factors, team factors, or factors of the working environment, served as an aid. For the cause-effect analysis, participants received a prepared Ishikawa diagram including the “bones”: patient, team, staff/individual, organization/management, and environment. The results of the group work were presented by the participants in a plenary.

In the patient involvement module, a 10-min input was first given on the topic of “communicating an adverse event,” followed by role play on the same topic. The input, based on the brochure “Reden ist Gold” (“Talking is Gold”) by the Patient Safety Action Alliance (Aktionsbündnis Patientensicherheit, 2017), gave an overview of the following points and questions: why communication with patients is important, especially after harm; when, where and with whom communication should happen after an incident; what should and may be said; what is the difference between an apology and an acknowledgment; and what patients want after an incident. The subsequent role play in communication after an adverse event was developed based on a case of CIRS Network Berlin. To facilitate the transfer of the training content into everyday work, the participating teams received two posters that could be hung in the ward room in a clearly visible place: each poster included the essentials of each of the topic areas of teamwork and error management. To remind participants of the contents of the topic area of patient involvement, postcards with key messages were sent to the wards on a monthly basis (e.g., “Your teach-back moment?”).

### 2.3. Data collection process

Each study site (Freiburg, Hamburg, Bonn) was responsible for recruiting at least 12 wards with 120 participants, so that a total of at least 36 different wards with 360 participants had agreed to participate. The inclusion criteria were: inpatient care teams (1) with at least 10 members, and (2) with an interprofessional composition. We excluded emergency and intensive care due to high regimentations and standardized procedures in teamwork. Furthermore, we excluded pediatrics, because patient involvement would not be comparable to other wards. A local study coordinator per ward (mostly ward manager) supported the research team regarding staff recruitment and data collection at their ward. The following inclusion criteria for study participants were applied: (1) member of an interprofessional inpatient care team (e.g., physician, nurse, therapist), (2) at least 18 years old, and (3) fluent in German.

Data were collected via online or paper-pencil questionnaire according to participants’ preferences. Prior to the start of data collection, the local study coordinators received an Excel list containing so-called one-time passwords (individual access codes) and the URL link to the online survey tool. They transmitted the access data (URL link and passwords) in-house to the participants via email. The stored data set contained a participant number corresponding to the access code (but no contact data) and was thus pseudonymized. This approach allowed data from all three data collection periods to be matched. If participants preferred paper-pencil questionnaires, the local study coordinator received the questionnaires marked with a version number as well as the data protection concept. The version number made it possible to track which wards were assigned to which group. The local study coordinator printed out the questionnaires and the data protection concept and distributed them to the participants. Completed questionnaires were collected in locked boxes. At the end, the questionnaires were entered manually into the online survey platform UniPark by researchers of the corresponding study site, so that all data were available in electronic form.

To increase the response rate in the three data collecting periods, multiple reminders following the Total Design Method of Dillman (2000) were sent to the participants. After sending an initial invitation to the survey, the participants received a first reminder to participate within 2 weeks. Within another 2 weeks, a second reminder containing the link to the questionnaire was sent. For data protection reasons and to prevent participants from recognizing those who had already answered the questionnaire, all thank-you letters and reminders were sent to all potential respondents by e-mail.

For the in-person training, the study coordinators in the hospitals recruited the participants.

The feasibility of eLearning was evaluated using an online questionnaire, whereas a questionnaire for evaluating the interprofessional in-person training was distributed directly after the training. The problem-focused interviews were conducted between June and October 2019. Interview appointments were requested electronically and confirmed via telephone or email. They were organized together with the study coordinators on the wards and held either face-to-face at the wards or over the phone.

### 2.4. Data analysis

The collected pilot test data was analyzed using descriptive and inferential statistical analyses to describe the sample and test the effectiveness of the interventions according to a modified intention-to-treat approach. These analyses included all participants providing data for at least one measurement and for at least one of the three measurement time points, randomizing all participant data at the ward level. Missing values were considered missing at random, dependent on information in the model, and accounted for using mixed models. The mixed models were calculated by including the intervention group (tree-level factor), time of measurement (three-level factor), interaction between the intervention group and time of measurement, sex (two-level factor), age (continuous variable), occupation (two-level factor), and fixed effects. Ward membership was modeled as having a random effect on the intercept. Statistical significance was set at *p* < 0.05. As the study was a pilot, no adjustment was made for multiple testing. Intracluster correlation coefficients were calculated to determine the proportion of the total variance that could be explained by ward affiliation. The standardized effect sizes (Cohen’s *d*) for pairwise group comparisons were calculated by dividing the estimated mean differences by the pooled observed standard deviations. To achieve robustness of the findings, a sensitivity analysis was performed with a subsample of participants from whom pre- and post-measurement data were available. Data were analyzed using IBM SPSS version 26 (IBM Corp., Armonk, NY, USA).

The questionnaires used to measure the feasibility of the interventions were analyzed using descriptive statistics. The interview data were transcribed externally according to the rules of Dresing and Pehl (2015). They were analyzed based on structured content analysis (Mayring, 2022) and used exploratively to cross-check the quantitative data. The categories were deductively derived. For subsequent data analysis, MAXQDA Plus 12 (VERBI Software Company) was used.

### 2.5. Ethics Statement and registration of the study

The project was approved by the ethics commissions at three study sites (Albert-Ludwigs-University of Freiburg: 4/16_170397, Friedrich-Wilhelm-University of Bonn: 329/17, Medical Association of Hamburg: MC-298/17). Participation was voluntary for the wards and team members. Consent for participation was obtained in written form. The study was registered in the German Register of Clinical Trials (DRKS-ID: DRKS00012818) on August 8, 2017.

## 3. Results

### 3.1. Pilot-testing

The study included at the beginning 39 interprofessional teams (mainly nurses and physicians) of different wards (ear, nose and throat wards; surgical wards; internal medicine wards; urology wards; gynecology wards; hematology wards; neurology wards; cardiology wards; orthopedic wards; psychosomatic wards) in 13 German hospitals. Participant characteristics are reported in Table 1. The majority of participants were female and were not older than 40 years of age. Most were nurses with several years of work experience.

**TABLE 1:** Description of the sample (*N* = 249).

	Included in the analysis					
	**IG1 eLearning (*n* = 139)**		**IG2 blended learning (*n* = 118)**		**WCG (*n* = 172)**	
	***N***	**%**	***N***	**%**	***N***	**%**
**Gender**						
Female	95	68	79	67	139	81
Male	44	32	39	33	33	19
**Age**						
≤ 30 years	48	35	44	37	43	25
31–40 years	53	38	26	22	53	31
41–50 years	21	15	27	23	35	20
> 50 years	17	12	21	18	41	24
**Profession**						
Physician	34	25	42	36	38	22
Nurses	99	71	70	59	109	63
Others	6	4	6	5	24	14
**Leading position**						
Yes	30	22	31	26	25	15
No	108	78	81	69	147	85
**Duration of hospital affiliation**						
< 3 months	4	3	3	3	3	2
> 3 months < 1 year	12	9	17	14	15	9
1–5 years	51	36	49	42	47	27
> 5 years	71	51	49	42	107	62
**Duration of occupational affiliation**						
< 3 months	5	4	2	2	2	1
> 3 months < 1 year	9	5	8	7	8	5
1–5 years	33	24	36	31	33	19
> 5 years	92	66	72	61	129	75

**FIGURE 3:**
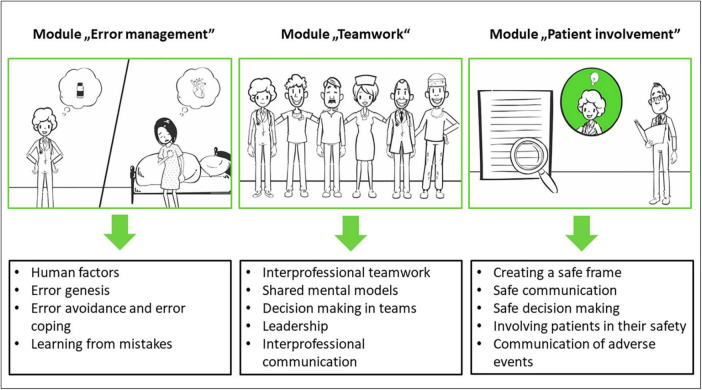
Modules of the eLearning (taken from the study protocol, Dinius et al., 2019).

A total of 846 individuals were randomly allocated to one of the three study arms (see Figure 3). The participation rate in interventions was 24.2% the blended learning group (IG 2) and 2.7% in the eLearning group (IG1: Out of 846 persons data from 335 persons were collected at baseline (response rate 39.6%), 191 of 846 persons (22.6%) at 12 weeks post-intervention, and 98 of 846 persons (11.6%) at 24 weeks follow-up. A total of 429 of 846 persons (50.7%) were included in the modified intention-to-treat analysis (see Figure 3).

Table 2 presents the observed means and standard deviations of the outcomes. It is notable that between-group differences at baseline often exceed the average within-group changes across time, suggesting a limited capacity of the cluster-randomization procedure to ensure balanced groups. The SJT for error management had a significant ceiling effect.

**TABLE 2:** Observed values for outcomes.

	IG1 (eLearning)			IG2 (blended learning)			WCG (waiting control group)		
	***N***	***M***	**SD**	***N***	***M***	**SD**	***N***	***M***	**SD**
**Subjectively perceived patient safety (min-max)**									
Pre-intervention	101	3.36	0.73	98	3.51	0.65	126	3.36	0.72
Post-intervention	38	3.29	0.90	54	3.67	0.51	92	3.49	0.64
Follow-up^2^	26	3.46	0.90	25	3.72	0.54	45	3.51	0.73
**SJT teamwork (0–100)**									
Pre-intervention	91	55.49	24.05	93	60.48	24.08	119	54.50	26.47
Post-intervention^1^	31	56.45	27.84	47	67.77	23.45	82	55.85	28.27
Follow-up^2^	20	49.50	20.19	19	48.42	21.54	38	42.63	22.17
**SJT error management (0–100)**									
Pre-intervention	95	90.74	13.43	94	91.22	15.29	120	91.21	14.71
Post-intervention^1^	32	89.69	9.75	49	88.27	16.85	86	90.64	13.17
Follow-up^2^	22	86.14	20.06	20	91.25	12.13	39	91.79	10.85
**SJT patient participation (0–100)**									
Pre-intervention	95	72.11	21.25	96	78.54	18.82	122	73.07	21.76
Post-intervention^1^	31	71.61	25.64	49	73.67	26.24	87	71.67	24.67
Follow-up^2^	19	69.21	20.77	20	79.50	19.73	39	81.15	15.71

^1^After 12 weeks. ^2^After 24 weeks. SJT, situational judgment test.

Table 3 shows the results of effectiveness testing via linear mixed modeling. After 12 weeks, participants with IG2 showed significantly higher teamwork in the SJT than participants in the WCG (*p* = 0.03, *d* = 0.42). No other statistically significant between-group differences were observed after 12 weeks. After 24 weeks, the IG2 group was significantly superior to IG1 (*p* = 0.01, *d* = 0.90), but not the WCG, which was also statistically significantly superior to IG1 (*p* = 0.01, *d* = −0.72). No other statistically significant between-group differences were observed. In the global tests, the mean changes across time were not statistically different between groups.

**TABLE 3:** Results of the pilot test of effectiveness.

	Blended learning vs. eLearning				Blended learning vs. waiting control group				eLearning vs. waiting control group				
	**Adj. effect**	**95% CI**	***p***	**Stand. effect *d***	**Adj. effect**	**95% CI**	***p***	**Stand. effect *d***	**Adj. effect**	**95% CI**	***p***	**Stand. effect *d***	**Global effect *p***
**Subjectively perceived patient safety (min-max)**													0.27
Post- intervention	0.34	−0.06 to 0.75	0.09	0.51	0.08	−0.29 to 0.46	0.64	0.12	−0.26	−0.64 to 0.12	0.17	−0.39	
Follow up	0.07	−0.37 to 0.50	0.76	0.10	0.07	−0.34 to 0.48	0.73	0.10	0.001	−0.40 to 0.40	0.99	0.001	
**Situational judgment—teamwork (0–100)**													0.71
Post-intervention	9.86	−2.60 to 22.31	0.12	0.37	11.29	1.32 to 21.26	0.03	0.42	1.43	−9.96 to 12.82	0.80	0.05	
Follow-up	−1.24	−15.10 to 12.62	0.86	−0.06	4.19	−8.05 to 16.43	0.50	0.20	5.43	−6.47 to 17.33	0.37	0.26	
**Situational judgment—error management (0–100)**													0.48
Post-intervention	−1.35	−6.92 to 4.21	0.63	−0.10	−1.62	−6.15 to 2.91	0.63	−0.12	−0.27	−5.38 to 4.84	0.92	−0.02	
Follow up	5.12	−3.68 to 13.92	0.25	0.37	1.29	−6.33 to 8.90	0.74	0.09	−3.84	−11.52 to 3.84	0.32	0.27	
**Situational judgment—patient involvement (0–100)**													0.13
Post-intervention	5.53	−5.90 to 16.97	0.34	0.22	4.63	−4.70 to 13.97	0.33	0.18	−0.90	−11.43 to 9.64	0.87	−0.04	
Follow up	16.13	4.67 to 27.59	0.01	0.90	3.19	−6.68 to 13.06	0.52	0.18	−12.94	−23.16 to −2.72	0.01	−0.72	

Adj. effect, mean difference in estimated marginal means; CI, confidence interval; Stand. Effect d, Cohen’s d; global effect *p*, *p*-value of the F-test of the group × time interaction examining whether mean changes across time were different between the groups.

Sensitivity analysis did not show statistically significant differences between the groups at the two measurement time points for any of the primary outcomes.

Intracluster correlation coefficients were calculated to determine the variance between wards. The proportion of variance explained by ward affiliation for the primary outcomes ranged from 0.0 (SJT teamwork) to 0.14 (subjectively perceived patient safety) at pre-assessment; from 0.05 (SJT teamwork) to 0.23 (subjectively perceived patient safety) at post- assessment; and from 0.05 (SJT teamwork) to 0.43 (SJT error management) at follow-up-assessment. The substantial contribution of ward affiliation to the overall variance sometimes supported the cluster-randomized approach and the approach of examining patient safety at the ward level.

### 3.2. Feasibility

#### 3.2.1. Feasibility of eLearning

Overall, adherence to eLearning participation was low despite the possibility of completing it independently of time and location. In both intervention groups, a total of 491 people were invited for eLearning and were entered into the system (IG1: *N* = 291, IG2: *N* = 200), of which *N* = 103 (IG1: *N* = 43, IG2: *N* = 60) opted in. This corresponds to a response rate of 20.98%. Due to stipulations in the ethics application, no information can be provided on the number of participants who completed eLearning in full.

The online questionnaire on eLearning was answered by 16 participants after completing the program (response rate 15.53%), and data was analyzed descriptively and exploratively. Participants rated eLearning using seven items on a scale of 1 (strongly disagree) to 5 (strongly agree). Table 4 presents the descriptive statistics for the individual items.

**TABLE 4:** Descriptive evaluation of the eLearning.

Item	Median (range)
The content of the eLearning was comprehensible.	4.5 (1)
The different forms of presentation (video, text, interaction) contributed to a better understanding of the content.	4.5 (1)
The eLearning was easily completed in the allotted time frame (60 min for each of the three topics: teamwork, error management, and patient involvement).	4.0 (3)
The eLearning was easy to use.	4.0 (2)
I liked the design of the eLearning.	4.0 (3)
I would use the eLearning again if I would refresh my knowledge in the topics of teamwork, error management, and/or patient involvement.	4.0 (5)
I would recommend the eLearning to colleagues who want to educate themselves in patient safety.	4.0 (5)

All items are rated from 1 (strongly disagree) to 5 (strongly agree).

Participants rated the comprehensibility of the content and the fact that the different forms of presentation contributed best to the understanding of the content. The design of eLearning was evaluated as the worst (see Table 4).

Additionally, using the German school grading system (1 = very good, 2 = good, 3 = satisfactory, 4 = sufficient, 5 = poor, 6 = deficient), participants awarded the program with an overall average grade of 2.13 (good).

#### 3.2.2. Feasibility of the interprofessional in-person training

For in-person training, 232 invitations were sent to 10 wards of IG2, of which 59 participants took part in the in-person training. The response rate was 25.43%.

A paper-based questionnaire on in-person training was completed by 57 participants (response rate: 96.61%). The item “The presenters were well prepared” received the highest ratings, while the item “The eLearning prepared me well for the in-person training” received the lowest ratings. Overall, the interprofessional in-person training was evaluated as good (Table 5).

**TABLE 5:** Descriptive evaluation of the in-person team training.

Item	Median (range)
The eLearning prepared me well for the in-person training.	4.0 (3)
The in-person training complemented the eLearning well.	5.0 (3)
The in-person training picked up relevant content from the eLearning.	5.0 (3)
The teaching methods used in the in-person training (e.g., video analysis, role play) are well suited for understanding the content.	5.0 (4)
The in-person training encouraged critical thinking about patient safety.	5.0 (3)
The in-person training taught me useful behavior that I will adopt in my daily work.	5.0 (3)
The in-person training had a positive impact on my team.	4.0 (3)
The amount of work required for in-person training is commensurate with the benefits.	4.0 (3)
The trainers were well prepared.	5.0 (3)

Items 1 to 7: Scale of 1 (strongly disagree) to 5 (strongly agree).

Additionally, using the German school grading system (1 = very good, 2 = good, 3 = satisfactory, 4 = sufficient, 5 = poor, 6 = deficient) to evaluate in-person training in total, it was rated by the participants with an average grade of 1.7 (good).

### 3.3. Qualitative results regarding feasibility

In addition, from the results of 30 problem-focused individual interviews, further indicators regarding the feasibility of the interventions were identified. Facilitating factors and barriers to the successful implementation of the intervention in the daily work routine were explored. Interviews were conducted between June and October 2019, and lasted 20 min on average. The sample consisted mainly of physicians and nurses, with most of them having less than 10 years of professional experience in their job and no leading position. The following categories were applied: user-friendliness, barriers, and facilitators.

The eLearning was described as well structured (“*It was well structured, articulated.*”, “*Structurally it was well done.*”), clear, realistic, and understandable, (“*So that it was realistic and easy to understand.*”, “*Basically you couldn’t go wrong.*”). The visual design was assessed as appealing (“*The design was good and clear.*”). Participants particularly emphasized the variety of task formats, visual presentation of the content, and videos used (“*The videos and the pictorial representation–I really liked that.*”). The processing time is sometimes too long (“*But I found that I was taking too much time. It was just too long for me.*”). The difficulty level was described as appropriate, and tasks and answer options as understandable (“*In terms of difficulty, I actually found it quite ok.*”).

Participants stated that they had difficulties integrating eLearning into their daily work routine. Implementation during working hours on the wards was hardly possible due to lack of time and interruptions (“*So at work [it] was just not possible for me*,” “*A point that is relatively difficult when you try to do it on the ward, you don’t have any peace and quiet.*”, “*Because when the patients see me, yes, they always talk to me*”). The possibility of conducting eLearning in sections was mentioned as being positive for feasibility (“*You are flexible, you can schedule it yourself, when, how, where, what. You don’t have to do everything at once.”, “However, I then simply did it in sections, then it was easier to do.*”). The reminder to carry out eLearning by study coordinators (“*I also had to […] be reminded again and again by Ms X, which simply helped me a lot*”), and the offer of some hospital wards to participate in eLearning credited as working time were experienced as beneficial (“*And we got time off work for the training, so we were sort of released from work.*”). On the other hand, a lack of technical equipment (lack of computer workstations), time pressure, and quiet in the daily work routine during the completion of eLearning, and IT security standards had a hindering effect on participation (“*Unfortunately, I couldn’t integrate it at all because I couldn’t open it here in my PC.*”).

Participants stated that they used eLearning primarily for knowledge activation and self-reflection (“*I found it very exciting because you can question yourself, but you probably wouldn’t go into the situation or question on your own.*”, “*You reflect in a completely different way.*”, “*And I think I’ve questioned myself a lot more now in relation to the patient.*”) and that the subsequent in-person training served to apply this knowledge.

Overall, participation adherence in eLearning was low. Additionally, participants reported that the learning effect of blended learning was higher than that of eLearning alone because of practical testing and the opportunity for interprofessional exchange during in-person training.

Moreover, it led to greater learning through hands-on testing (“*This group work in particular, where you can then exchange ideas with one another, where you can also talk to the lecturers, makes more sense, or I learn or I take more with me.*”). The ability to ask questions directly has been highlighted (“*I found the in-person training easier to understand because I could ask directly if I didn’t understand something.*”). Participants emphasized that the training content made them more aware of their everyday work, that their sense of safety was enhanced by the application of learned communication strategies, and that awareness of patient safety was increased (“*[.] I feel safer now that I [.] have a scheme like this that I can use to shimmy along*,” “*[…] you look at certain situations or certain things from a different perspective and question things differently.*”, “*Afterward we talked about it in the team and it really became clear to everyone how important communication is and that you talk to each other and that you take the initiative and in such situations ask clear questions, give clear answers and so on.*”). In addition, the participants reported that their courage to approach colleagues about mistakes increased. “*Since then, I’ve been taking a closer look. That’s because the program has brought it to the foremind again.*”, “*[…] and we could talk more openly about problems and mistakes now*,” “*[.] as the head of the ward, I observed that the participants returned to the ward with great commitment […] and have spoken more consciously with colleagues about mistakes or even approached colleagues.*”).

Facilitation of training participation for in-person training was done through the exemption of teams from their shifts in the ward. Participants particularly emphasized the comprehensible and motivating didactic delivery of the content and indicated a lot of enjoyment and an open atmosphere when working on the tasks in groups and plenary sessions (“*The group work was fun for us.*”, “*Well, that was really, really good, very pleasant atmosphere, I have to say. The learning material that was presented to us was really conveyed in a way that we understood it straight away, and that we were able to get involved very well.*”). However, the participation of all professionals from one ward was compromised because of conflicting schedules (“*We couldn’t all attend together because our schedules are different.*”).

## 4. Discussion

The aim of the study was to pilot the effectiveness and feasibility of interventions. To this end, a cluster-randomized study on the effectiveness of the training program and a descriptive-explorative study regarding its feasibility were conducted. While the cluster-randomized study on the effectiveness of the interventions did not show consistent differences between the groups or a clear pattern in the different outcomes, the formative study analysis (feasibility study) resulted in a high level of acceptance and stressed the importance of daily work for participating in the intervention. At present, there is insufficient evidence to support our hypothesis that IPTP improve safety-related behavior with regard to teamwork, error management, and patient involvement as well as subjectively perceived patient safety. We can only assume why no empirical effects were found in this study. The main assumptions about this are as follows. First both interventions are not effective because they are carried out once (there are no booster sessions) and they are too short. Second, if the interventions are not tested as planned because of the low response rate, they will not have an effect. Third, the outcome measures did not capture the effects of the interventions reliably and validly. A possible solution would be to check the methodological quality of the SJT. Regarding the implementation of the in-person intervention, a trainer model should be considered in follow-up studies to test the sustainability of the in-person intervention and organizational learning in clinics. Managerial support is especially important; therefore, a step-by-step approach could be a solution for the better utilization of both interventions.

Overall, the professionals participated in the interventions rated the intervention as positive and described the content as extremely helpful for their everyday work. The insights gained in the formative evaluation include the following. First, participating wards in the eLearning intervention should be provided with technical equipment (tablet computers). Second, both eLearning and interprofessional in-person training should explicitly consider working schedules, particularly when seeking to train the entire team. Third, at least two different dates should be offered for each in-person training for employees who otherwise could not take part due to conflicting schedules. Fourth, the importance of participation should be communicated, particularly to ward managers who have a role model function, so that they can participate in training and encourage their employees to participate.

Amaral et al. (2023) summarized studies on the effectiveness of patient safety training, stating that there are still few studies that test patient safety training programs. Most studies focus on the development of training programs and do not provide evaluation data. Furthermore, training programs are mostly designed for special healthcare professional groups, and the interprofessional aspect is not explicitly considered (Dinius et al., 2019). Amiri et al. (2018) conducted a RCT (randomized controlled trial, RCT) and showed a significant improvement in safety culture. However, the dimensions of non-punitive responses to errors reported as adverse events did not improve, indicating that additional actions are necessary (Amiri et al., 2018). Overall, there is moderate to high-quality evidence that team training has a positive impact on healthcare team processes and patient outcomes (Weaver et al., 2014). Several studies have reported positive outcomes, such as patient-centered communication, improved clinical outcomes, collaborative practice, reduction of clinical error rates, and improved team behavior (Reeves et al., 2013). A current systematic review and meta-analysis (Agbar et al., 2023) including 16 studies and 6,559 participants from healthcare professional staff showed that the interventions have a positive effect on safety culture. However, the interventions varied between the studies and there was a significant heterogeneity among the studies assessing patient safety culture (Agbar et al., 2023, p. 1471). Furthermore, the effects were no longer significant after the exclusion of studies with low quality scores (Agbar et al., 2023). The integrative review of Lee et al. (2022) identified also “non-significant and inconsistent relationships between safety culture and patient safety and quality of care outcomes” (p. 279). Several different factors could contribute to these inconsistent and non-significant results, such as the lack of a theoretical framework, inconsistent outcome criteria, missing validity of the instruments, etc (Lee et al., 2022). Overall, the study situation is inconsistent, so that the present study fits into the previous picture. Interprofessional training has become increasingly common in recent years. The KOMPAS training program was explicitly interprofessional, but in some clinics, only nurses participated. In addition to the lack of doctors, the participation of health professionals in the training sessions and surveys was very low. The dropout rate was 22.5%. The reasons for dropout included refusal to participate in part of the contacts and difficult accessibility. The healthcare professionals struggled with the lack of support, lack of resources, time constraints, or conflicting priorities in their clinics that hindered an adequate training implementation. Furthermore, the evaluation methods used rely on self-report measures that do not capture the true impact.

As most interventions include narrative reviews on the nature of interprofessional interventions to promote patient safety by Reeves et al. (2017), our intervention is an educational intervention to address individuals’ skills and behaviors. The key principles (human factors, systems approach, teamwork, communication, and error reporting) of our patient safety training are comparable to other training programs (Lee et al., 2022).

To measure the feasibility and effects of the intervention, we conducted surveys, as most of the reported studies in the narrative review. However, in contrast to the vast majority (86 out of 89) of the studies included in the review, we applied a mixed-methods approach and conducted qualitative interviews. Most studies have concentrated on nurses and physicians in acute care, as in our study. A dearth of studies has reported changes in safety behaviors, which we aimed to measure using SJTs. The choice of SJTs as our main outcome measures could be a crucial aspect of the inconsistent and non-significant results.

### 4.1. Limitations

The moderate response rates and exclusion of pediatric, emergency, and intensive care wards limit the generalizability of the study results only to the target population of individuals. Concurrently, the inclusion of hospitals from different regions in Germany may strengthen the generalizability of the results to the populations of targeted organizations. As participation was voluntary, we must assume a selection bias because we cannot exclude that we mainly reached motivated and well-functioning interprofessional teams. A serious limitation was the low proportion of invited participants who received the interventions. In addition, the internal validity of the pilot effectiveness test is likely to be limited by the imperfectly balanced groups at baseline, missing controls of possible co-interventions, and the high attrition rate across measurements. Concerning the instrument, we developed specific SJTs based on current literature and experts’ opinions on the primary outcomes. However, these are not psychometrically validated instruments. Patient safety is measured using only one ordinal-scale item, which possibly restricts the variability of participants’ responses. Future studies should use objectively measured outcomes to assess patient safety.

Moreover, it is worth considering the appropriateness of utilizing RCTs for assessing these interventions. There is a significant challenge evaluating changes and improvement in practice at this level. We encountered several challenges during the study. Given that both the tested interventions and the circumstances can be considered “complex,” it is possible that other (e.g., multi-level designs, sequential designs, non-randomized designs) might have suited better to our research aims than a RCT (Skivington et al., 2021).

The curriculum was feasible and judged as relevant and useful, but the participation rate was very low and we had a high drop-out rate. This can be due to the wrong implementation strategy in a very complex setting with a high workload. This means that an actual study with the developed intervention was not conducted. For further studies, the design and implementation of the intervention in particular must be redesigned with a comprehensive participatory research approach.

### 4.2. Future development

Future studies should further examine the effectiveness of the intervention within a confirmatory study after the implementation of the intervention has been further developed based on the current results. To anchor the knowledge comprehensively in clinics, a multi-stage procedure should be chosen, starting with clinic and ward managers. This approach is intended to communicate the importance of participating in such an intervention to ward managers, who function as role models, so that they can disseminate on the topic top-down to their staff. As a result, improved adherence to participation in the intervention and evaluation could be encouraged.

To implement the intervention in a sustainable way, the wards should offer booster sessions after the intervention. Furthermore, individual and group coaching sessions are proven methods to increase participants’ self-efficacy and further steps for evaluating the training program include consideration of the patients’ perspectives and the organizational preconditions. To consider these long-term outcomes, longitudinal mixed methods and multilevel studies are required.

## Data availability statement

The raw data supporting the conclusions of this article will be made available by the authors, without undue reservation.

## Ethics statement

The studies involving humans were approved by the Albert-Ludwigs-University of Freiburg: 4/16_170397, Friedrich-Wilhelm-University of Bonn: 329/17, Medical Association of Hamburg: MC-298/17. The studies were conducted in accordance with the local legislation and institutional requirements. The participants provided their written informed consent to participate in this study.

## Author contributions

LK designed the pilot effectiveness trial, performed data analysis, and interpreted the results. MK developed the initial article, whereas JD, NE, LH, CB, AH, and SP-H read the manuscript critically and provided relevant corrections, additions, and comments. All the authors established the project application, collectively provided the basis for this study, critically reviewed, and approved the final manuscript.

## Footnotes

1
https://www.teamstepps.de/

2
https://wb-ilias.uni-freiburg.de/ilias.php?ref_d=291255&cmd=render&cmdclass=ilrepositorygui&cmdnode=111&baseclass=ilrepositorygui
